# A293 PREVALENCE OF METABOLIC ASSOCIATED STEATOTIC LIVER DISEASE AND ITS IMPACT ON ADVERSE OUTCOMES IN PRIMARY BILIARY CHOLANGITIS PATIENTS

**DOI:** 10.1093/jcag/gwad061.293

**Published:** 2024-02-14

**Authors:** R Kaviani, Y Wong, D Shreekumar, A Mason, A Montano Loza, E Lytvyak

**Affiliations:** Gastroenterology, University of Alberta, Edmonton, AB, Canada; Gastroenterology, University of Alberta, Edmonton, AB, Canada; Gastroenterology, University of Alberta, Edmonton, AB, Canada; Gastroenterology, University of Alberta, Edmonton, AB, Canada; Gastroenterology, University of Alberta, Edmonton, AB, Canada; Gastroenterology, University of Alberta, Edmonton, AB, Canada

## Abstract

**Background:**

Metabolic associated steatotic liver disease (MASLD) is highly prevalent among Canadians, thus making coexistence with other liver diseases inevitable. Primary biliary cholangitis (PBC) is the most common autoimmune liver disease diagnosed in one out of 1,000 women above 40. The impact of concomitant MASLD on liver outcomes in patients with PBC is unclear.

**Aims:**

We aimed to determine the prevalence of MASLD among patients with PBC and the association between concomitant liver disease and adverse liver outcomes.

**Methods:**

PBC patients diagnosed between 1984 and 2023 and followed up at the University of Alberta with vibration-controlled transient elastography data available were included. MASLD was diagnosed if the Controlled Attenuation Parameter ≥288 dB/min with at least one cardio-metabolic criteria, based on elevated body mass index, blood pressure, and abnormal glycemic and lipid tests. Proportions were compared using chi-square, medians – Mann-Whitney test. We utilized a Cox hazards regression model to calculate hazard ratios and determine associations between MASLD and the development of cirrhosis or decompensation, need for liver transplant, or death.

**Results:**

A total of 115 patients (87.0% females, age at diagnosis 52.3±11.5 years) followed over a median duration of 11.0 [range 0.1-39.7] years) were included. The prevalence of MASLD (33.0% [n=38]) was higher in females than males (35.0%[n=35] vs. 20.0%[n=3]; p=0.249). The prevalence of cirrhosis at diagnosis was twice as high among PBC patients with MASLD than those without (26.3%[n=10] vs. 13.0%[n=10]; p=0.076). No difference was observed in the frequency of decompensation at diagnosis (8.8%[n=3] vs. 6.3%[n=4]; p=0.638). Cirrhosis frequency (50.0% vs. 50.6%; p=0.948) and decompensation (38.2% vs.37.1%; p=0.912) over the course of the disease were similar between PBC patients with and without MASLD. There was no association between MASLD and development of cirrhosis (HR0.51, 95%CI 0.23-1.13; p=0.095), decompensation (HR0.81, 95%CI 0.37-1.76; p=0.596), liver transplantation (HR0.72, 95%CI 0.19-2.72; p=0.628), or death (HR0.69, 95%CI 0.22-2.16; p=0.519). PBC patients with MASLD have comparable event-free (median 10.7 [3.1-30.8] vs. 10.7 [0.1-23.3] years; p=0.684) and overall survival (median 11.3 [3.2-39.7 vs. 10.7 [0.1-23.3] years; p=0.456).

**Conclusions:**

Every third patient with PBC had MASLD, which tended to be more prevalent among females. We did not observe any significant differences in the development of adverse liver outcomes between patients with PBC and concurrent MASLD in comparison to those without. Studies with larger sample sizes are necessary to further explore the potential impact of MASLD on liver outcomes.

**Funding Agencies:**

None

